# Plasma amino-acid determinations by reversed-phase HPLC: Improvement of the orthophthalaldehyde method and comparison with ion exchange chromatography

**DOI:** 10.1155/S1463924692000270

**Published:** 1992

**Authors:** Frédéric Ziegler, Jacques Le Boucher, Colette Coudray-Lucas, Luc Cynober

**Affiliations:** 1 Laboratoire de Biochimie UER des Sciences Pharmaceutiques et Biologiques Université Paris XI, Chatenay 92296 France; 2 Laboratoire de Biochimie UER des Sciences Pharmaceutiques et Biologiques 5 rue J. B. Clément Chatenay-Malabry Cedex 92296 France

## Abstract

Reversed-phase high performance liquid chromatographic (RPHPLC) determination of amino-acids with on-line pre-column ortho-phthalaldehyde (OPA) derivatization and fluorescence detection is rapid and sensitive. However, high-performance ionexchange chromatography (HP-IEC) with post-column ninhydrine reaction is the most widely used amino-acid (AA) assay for biological samples. These two methods have been compared for the determination of individual plasma AA concentrations.

An excellent correlation (p ≤ 0.003) was found between the results given by an RP-HPLC system (Varian) and a classical AA autoanalyser (Biotronik LC 5001). Most AA concentrations were similar with the two methods, but a paired t-test showed slight differences for 13 AA. The within-batch reproducibility of the RPHPLC method was comparable to that of the HP-IEC method. The analysis was about three times faster with RP-HPLC, and sensitivity was l00-fold better. However, aspartic acid, proline and cysteine were not identified by the RP-HPLC method, while the tryptophan quantification is possible.

RP-HPLC with automated pre-column OPA derivatization is clearly a suitable alternative for assaying physiological AA and may be particularly useful for AA present at low concentrations (free tryptophan, plasma 3-methylhistidine).


Journal of Automatic Chemistry, Vol. 14, No. 4 (July-August 1992), pp. 145-149

### Plasma amino-acid determinations by reversed-phase HPLC: Improvement of the orthophthalaldehyde method and comparison with ion exchange chromatography

**Frédéric Ziegler, Jacques Le Boucher, Colette Coudray-Lucas and Luc Cynober**

*Laboratoire de Biochimie, UER des Sciences, Pharmaceutiques et Biologiques, Université Paris XI, 92296 Chatenay, France*

*Reversed-phase high performance liquid chromatographic (RP-HPLC) determination of amino-acids with on-line pre-column ortho-phthalaldehyde (OPA) derivatization and fluorescence detection is rapid and sensitive. However, high-performance ion-exchange chromatography (HP-IEC) with post-column ninhydrine reaction is the most widely used amino-acid (AA) assay for biological samples. These two methods have been compared for the determination of individual plasma AA concentrations.*

*An excellent correlation ($p \leq 0{\cdot}003$) was found between the results given by an RP-HPLC system (Varian) and a classical AA auto-analyser (Biotronik LC 5001). Most AA concentrations were similar with the two methods, but a paired t-test showed slight differences for 13 AA. The within-batch reproducibility of the RP-HPLC method was comparable to that of the HP-IEC method. The analysis was about three times faster with RP-HPLC, and sensitivity was 100-fold better. However, aspartic acid, proline and cysteine were not identified by the RP-HPLC method, while the tryptophan quantification is possible.*

*RP-HPLC with automated pre-column OPA derivatization is clearly a suitable alternative for assaying physiological AA and may be particularly useful for AA present at low concentrations (free tryptophan, plasma 3-methylhistidine).*

### Introduction

The most widely used method for amino-acid (AA) determination in biological fluids is high-performance ion-exchange chromatography (HP-IEC) [1,2]. However, despite considerable improvements [3–5], the run time is still lengthy and when ninhydrin-based detection is used, the method is poorly sensitive for AAs present at low concentrations, such as aspartic acid, citrulline, methionine and tryptophan [6].

Reversed-phase high-performance liquid chromatography (RP-HPLC) is an alternative method. Most RP-HPLC-based assays involve pre-column derivatization with various agents such as ortho-phthalaldehyde (OPA) [7], phenylisothiocyanate (PITC) [8], 9-fluorenylmethylchloroformate (FMOC) [9], and 4-fluoro-7-nitrobenzo-2-oxa-1,3-diazole (NBD-F) [10]. There is a general preference for OPA, given its simplicity of use, but, paradoxically, the speed of elution makes it difficult to separate the numerous AAs present in plasma. Several solutions to this problem have been proposed [11–13] differing by the nature of the buffers, the length of the column and/or the size of the solid-phase particles.

Another major problem with OPA derivatives is their instability, which means that the time between derivative formation and injection into the HPLC system must be carefully controlled [14]. An apparatus in which the mixing and sampling steps are automated should thus be suitable for routine (24-hour) OPA-based plasma AA analysis.

The performance of an OPA-based method derived from that of Fürst *et al.* [15] using the automatic Vista Varian system was evaluated and the results compared to those given by automated HP-IEC.

### Materials and methods

### *RP-HPLC method*

#### *Apparatus*

The Varian RP-HPLC system (Walnut Creek, USA) consists of a model-9090 autosampler coupled to a Vista 5500 liquid chromatograph, a model-2070 spectrofluorimetric detector (excitation wavelength 330 nm, emission wavelength 450 nm) and a model-4290 integrator. The chromatographic column was a Spherisorb ODS II [15 cm × 4·6 mm (ID)] packed with 3-μm particles and equipped with a 10 mm × 4·6 mm (ID) guard column (Société Française de Chromato Colonne, Neuilly-Plaisance, France).

#### *Buffers*

Two buffers (A and B) were prepared using ultrapure water generated with a Milli-Q system (Millipore, Molsheim, France). The chemicals were of analytical grade and the solvents of chromatographic grade. Buffer A contained 12·5 mM sodium phosphate and 0·5% tetrahydrofuran (THF, Merck) and was adjusted to pH

Correspondence to Dr L. Cynober, Laboratoire de Biochimie, UER des Sciences Pharmaceutiques et Biologiques, 5 rue J. B. Clément, 92296 Chatenay-Malabry Cedex, France.

0142-0453/92 $3.00 © 1992 Taylor & Francis Ltd.

7·2. Buffer B consisted of 12·5 mM sodium phosphate, methanol (Farmitalia Carlo Erba, Milano, Italia) and acetonitrile (Merck, Darmstadt, Germany), (50/35/15 by volume), adjusted to pH 7·2.

The buffers were filtered through 0·22 μm filters (Millipore) and directly delivered into the HPLC reservoirs (Ultraware System, Bioblock, Illkirch, France).

*Standard and sample preparation*

A 200 μM stock standard solution containing 28 physiological AAs was prepared using a commercial solution (AA-S-18, Sigma, La Verpillière, France); α-aminobutyric acid, asparagine, carnosine, citrulline, glutamine, 1-methylhistidine, 3-methylhistidine, ornithine, taurine and tryptophan (Sigma) were also added. Before analysis, the standard and samples were diluted (1/20) in buffer A containing norvaline (Sigma) as internal standard.

*OPA-3MPA reagent preparation*

50 mg of OPA (Sigma) were dissolved in 100 ml of potassium borate buffer (1·0 M, pH 10·4) (Varian). 100 μl of 3-mercaptopropionic acid (3MPA) was then added and the solution was filtered through a 0·22 μm filter (Millipore). The solution is stable for up to two days when kept in the dark at 4 °C under nitrogen.

*Autosampling process*

The autosampler automatically mixes the reagents, washes the tubing, and injects the mixtures into the analytical column; an autostart signal is transmitted to the pump and integrator programs.

The OPA-3MPA reagent, the samples and the diluting buffer (buffer A) were placed separately in different brown self-sealing microvials on the autosampler racks. 10 μl of OPA reagent are aspirated from the vial, and ejected into an empty self-sealing microvial; the tubing is then washed three times with buffer A, and 10 μl of the sample is added. After a further wash, 80 μl volume of buffer A are added. After mixing by three flux/reflux steps, 30 μl are aspirated through the 20 μl sample loop in the injection valve and injected into the analytical column. The tubing is washed nine times to prevent cross-contamination between the samples and reagents. The autosampling process starts 12 min before injection, while the pump program is still running. The OPA reagent and sample are in contact for precisely 3 min prior to the injection.

*Elution program and chromatographic conditions*

With a constant flow rate of 0·9 ml/min, the AA are eluted by linearly increasing the percentage of buffer B from 0 to 100% over 43 min. Buffer B is maintained at 100% for 2 min and then reduced to 0% in 2 min. The column is equilibrated with 100% buffer A for 8 min before the next sample injection (total run time 55 min).

At the flow rate used, the basal pressure is about 150 bars; during the analysis, it increases to about 250 bars in parallel with the increase in the viscosity of the mobile phase due to mixing of the buffers. Pressure returns to base-line after equilibration of the column.

*HP-IEC method*

The HP-IEC system used in this study consisted of a conventional integrated AA autoanalyser (Biotronik LC 5001, Munich, Germany) with postcolumn reaction with ninhydrine [16], and detection at both 440 and 570 nm. The data were collected using an SP 4290 integrator (Spectra Physics, San Jose, USA).

*Subjects*

Blood was drawn into heparinized tubes after an overnight fast from a forearm vein of 27 healthy subjects (age: 28 ± 5 years 15M, 12F). Plasma was immediately separated by centrifugation (4 °C), deproteinized with sulphosalicylic acid (SSA; 50 mg . $ml^{-1}$) and stored at −20 °C until analysis.

*Statistical analysis*

$\chi^2$ and Kolmogorov-Smirnov tests showed that plasma AA concentrations were normally distributed, in agreement with the literature [17]; Student's paired and unpaired t-tests were thus applied, using the PCSM Deltasoft program package (Grenoble, France).

Results are given as mean ± standard deviation (x ± SD).

### Results and discussion

In the conditions described above, the RP-HPLC method separated 24 AAs (including norvaline, the internal standard). Typical chromatograms for the standard and plasma AAs are shown in figure 1. The last OPA-3MPA derivative (lysine) was eluted in less than 40 min. The analysis of a single sample took 55 min with RP-HPLC and 141 min with HP-IEC.

The results of the analysis of plasma samples from 27 healthy volunteers by means of the two methods are summarized in table 1. The values were slightly different for 13 AA (paired t-test ≤ 0·05), but remained within the same range. Correlations between the results of the two methods are also shown in table 1. A very strong correlation was obtained for all the AAs analysed, with the exception of MET; as the plasma concentration of MET was the lowest of the AAs studied, the IEC method may have given an erroneous value as it is less sensitive than the OPA-3MPA method.

The repeatability of the assays was studied by performing ten consecutive measurements. The coefficient of variation (CV) for the RP-HPLC method ranged from 2·1% (ILE) to 5·7% (LYS), values similar to those of HP-IEC (from 1·9% [GLN] to 3·5% [HIS]). The CVs for RP-HPLC were much lower than those obtained recently by Fermo *et al.* [18], who used 2-mercaptoethanol rather than 3MPA, but it is known that this reagent forms less stable derivatives [12].

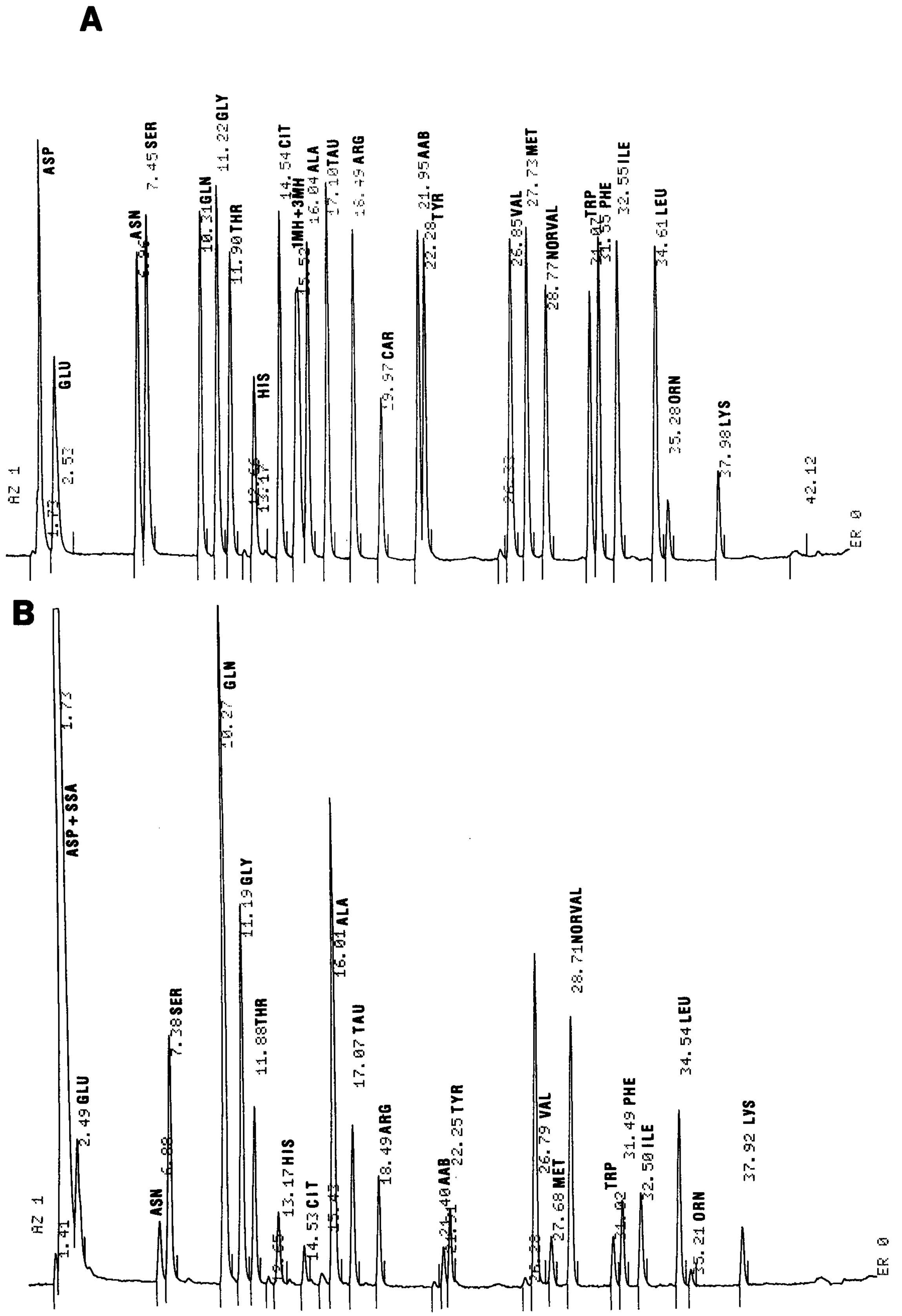

*Figure 1. Typical chromatograms of an (automated) OPA-3MPA derivatized (A) amino-acid standard and (B) human plasma. SSA = sulfosalicylic acid (deproteinizing agent), ASP = aspartic acid, GLU = glutamic acid, ASN = asparagine, SER = serine, GLN = glutamine, GLY = glycine, THR = threonine, HIS = histidine, CIT = citrulline, 1MH = 1-methylhistidine, 3MH = 3-methylhistidine, ALA = alanine, TAU = taurine, ARG = arginine, CAR = carnosine, AAB = α-aminobutyric acid, TYR = tyrosine, VAL = valine, MET = methionine, NORVAL = norvaline (internal standard), TRP = tryptophan, PHE = phenylalanine, ILE = isoleucine, LEU = leucine, ORN = ornithine, LYS = lysine.*

*Table 1. Correlations between RP-HPLC and HP-IEC results for plasma AA concentrations in healthy subjects.*

| Amino-acid (AA) | RP-HPLC x ± SD (μmol/l) ($N = 27$) | HP-IEC x ± SD (μmol/l) ($N = 27$) | Coefficient of correlation* between the two methods |
|---|---|---|---|
| GLU | 86 ± 74 | 67 ± 44 | 0·81 |
| ASN | 46 ± 7■ | 65 ± 10 | 0·90 |
| SER | 118 ± 15 | 119 ± 20 | 0·89 |
| GLN | 493 ± 95■ | 554 ± 113 | 0·95 |
| GLY | 221 ± 41 | 216 ± 47 | 0·95 |
| THR | 121 ± 22■ | 140 ± 27 | 0·95 |
| HIS | 109 ± 14■ | 91 ± 11 | 0·66 |
| CIT | 24 ± 5■ | 33 ± 7 | 0·60 |
| ALA | 340 ± 50■ | 358 ± 56 | 0·70 |
| TAU | 70 ± 11■ | 75 ± 11 | 0·80 |
| ARG | 82 ± 20 | 81 ± 21 | 0·93 |
| AAB | 22 ± 6■ | 28 ± 6 | 0·87 |
| TYR | 57 ± 13 | 55 ± 12 | 0·93 |
| VAL | 232 ± 40■ | 255 ± 36 | 0·96 |
| MET | 31 ± 5■ | 21 ± 4 | 0·55 |
| TRP | 38 ± 5 | — | — |
| PHE | 54 ± 7■ | 76 ± 9 | 0·75 |
| ILE | 65 ± 12 | 63 ± 13 | 0·96 |
| LEU | 124 ± 21 | 123 ± 21 | 0·95 |
| ORN | 50 ± 10■ | 65 ± 17 | 0·75 |
| LYS | 150 ± 27■ | 211 ± 28 | 0·88 |

■ $p \leq 0{\cdot}05$ versus HP-IEC (paired t-test).
* $p < 0{\cdot}001$ for all AAs except MET, $p < 0{\cdot}003$.

In comparison with HP-IEC, the OPA-3MPA method has the major advantage of having widespread applications because of its analytical versatility; in addition, it requires only two buffers rather than five.

The OPA derivatization method is simpler than other RP-HPLC methods since the derivatives are performed in aqueous conditions, and evaporation and heating steps are not required.

Tryptophan (TRP) was well separated and the fluorescence response of its derivative was comparable to that of aliphatic AA. TRP is more difficult to measure with HP-IEC because of its low sensitivity to ninhydrine and its relatively low plasma concentration (less than 50 μmol/l). RP-HPLC separation with OPA-3MPA pre-derivatization is thus useful for determining this essential AA.

Good overall analytical precision was obtained despite differences in derivative stability. The major reason is probably the reduced time between derivatization and injection that automation allows. Secondly, as stated above, the use of 3MPA instead of 2 mercaptoethanol, improves derivative stability [12]. Interactions between OPA-3MPA derivatives and the reversed-phase column result in a better peak resolution and stable retention times. Resolution was also improved by including tetrahydrofuran and methanol in buffers A and B, respectively. Base-line pressure is reduced and separation is preserved by shortening the column (15 cm instead of 25 cm) [12,15]. The additional automated dilution (1/10) before sampling reduces OPA-induced interference, resulting in a stable base-line.

The OPA-based RP-HPLC method does, however, have some of the disadvantages of other OPA methods, particularly the fact that imino-acids such as proline and hydroxyproline cannot be derived directly [3,4]. The cysteine derivative shows a low fluorescence intensity, but this can be overcome by, for example, pretreating the sample with iodoacetic acid [19]. ASP coelutes with SSA but this problem can be by-passed by using acetonitrile as the deproteinizing agent [20].

Finally, in the analytical conditions used here, 3-methylhistidine (3MH) coeluted with 1-methylhistidine (1MH). Plasma 3MH, a marker of myofibrillar catabolism assessed by measuring arterio-venous differences [21], can be measured specifically by simply changing the composition of buffer B [22], and adapting the elution program (not shown).

In conclusion, RP-HPLC with fluorescence detection of OPA-3MPA derivatives is a rapid and reliable method for the quantitative determination of free AAs in human plasma. Its greater sensitivity (100-fold) than HP-IEC with ninhydrine detection should make it applicable to measurements of AAs present at low concentrations in plasma (for example free TRP or 3MH).

### References

1. Moore, S., Spackman, D. H. and Stein W. H., *Analytical Chemistry,* **30** (1958), 1185.
2. Moodie, I. M., Shephard, G. S. and Labadarios, D., *Journal of High Resolution Chromatography,* **12** (1989), 509.
3. Roth, M., *Analytical Chemistry,* **43** (1971), 880.
4. Benson, J. R. and Hare, P. E., *Proceedings of the National Academy of Science (USA),* **72** (1975), 619.
5. Cynober, L., Coudray-Lucas, C., Ziegler, F. and Giboudeau, J., *Journal of Automatic Chemistry,* **7** (1985), 201.
6. Cynober, L., Ziegler, F., Coudray-Lucas, C., Chauffert, M. and Giboudeau, J., *Annales de Biologie Clinique,* **45** (1987), 537.
7. Turnell, D. C. and Cooper, J. D. H., *Clinical Chemistry,* **28** (1982), 527.
8. Biggs, H. G. and Gentilcore, L. J., *Clinical Chemistry,* **30** (1984), 581.
9. Einarsson, S., Josefson, B. and Lagerqvist, S., *Journal of Chromatography,* **282** (1983), 609.
10. Kotaniguchi, H., Kawakatsu, M., Toyo'oka, T. and Imai, K., *Journal of Chromatography,* **420** (1987), 141.
11. Fernstrom, M. H. and Fernstrom, J. D., *Life Sciences,* **29** (1981), 2119.
12. Godel, H., Graser, T., Foldi, P., Pfaender, P. and Furst, P., *Journal of Chromatography,* **297** (1984), 49.
13. Rajendra, W., *Journal of Liquid Chromatography,* **10** (1987), 941.
14. Garcia Alvarez-Coque, M. C., Medina Hernandez, M. J., Villa Nueva Camanas, R. M. and Mongay Fernandez, C., *Analytical Biochemistry,* **178** (1989), 1.
15. Furst, P., Pollack, L., Graser, T. A., Godel, H. and Stehle, P., *Journal of Chromatography,* **499** (1990), 557.
16. Spackman, D. H., Stein, W. H. and Moore, S., *Analytical Chemistry,* **30** (1958), 1190.

17. Cynober, L., Blonde, F., Nguyen-Dinh, F., Gerbert, D. and Giboudeau, J., *Annales de Biologie Clinique,* **41** (1983), 33.

18. Fermo, I., Devecchi, E., Diomede, L. and Paroni, R., *Journal of Chromatography,* **534** (1990), 23.

19. Cooper, J. D. H. and Turnell, D. C., *Journal of Chromatography,* **227** (1982), 158.

20. Uhe, A. M., Collier, G. R., McLennan, E. A., Tucker, D. J. and O'Dea, K., *Journal of Chromatography,* **564** (1991), 81.

21. Sjolin, J., Stjernström, H., Arturson, G., Andersson, E., Friman, G. and Larsson, J., *Metabolism,* **50** (1989), 1407.

22. Van Eijk, H. M. H., Deutz, N. E. P., Wagenmakers, A. J. M. and Soeters, P. B., *Clinical Chemistry,* **36** (1990), 556.

